# Microscopic Characterization and Efficiency Coefficient Evaluation of Modified Recycled Concrete Micropowder in Cementitious Materials

**DOI:** 10.3390/ma19112391

**Published:** 2026-06-03

**Authors:** Qiuyi Li, Pengfei Zhang, Mingxu Chen, Liang Wang, Gongbing Yue, Jinghua Yan, Chenyang Xu, Yuanxin Guo

**Affiliations:** 1 College of Civil Engineering & Architecture, Qingdao Agricultural University, Qingdao 266109, China; 2 State Key Laboratory of Silicate Materials for Architectures, Wuhan University of Technology, Wuhan 430070, China; 3 College of Civil Engineering, Hunan University, Changsha 410082, China

**Keywords:** efficiency coefficient, modified recycled micropowder, hydration characteristics, recycled silica-based mortar, mortar performance evaluation

## Abstract

To advance the development of sustainable buildings, this study investigates recycled cement-based materials. The core component of this material is concrete-based recycled micropowder (CRM), which is shaped and reinforced from recycled construction waste. It is then activated through high-temperature calcination to produce modified recycled micropowder (MRM), and the resulting changes in its properties are analyzed. X-ray diffraction, Brunauer–Emmett–Teller surface area, and hydration heat tests reveal that cementitious materials incorporating MRM800 contain more C-S-H and other hydration products, exhibit lower porosity, and demonstrate stronger hydration reactions. The results show that 800 °C is the optimal calcination temperature for CRM activation. For recycled silica-based mortar (RSM), the introduction of an efficiency coefficient (K_λ_) allows for a quantitative, scientific, and intuitive evaluation of the contributions of three admixtures, aiding in the optimization of the mix ratio. RSM with MRM showed improved performance, with compressive strength ranging from 24.3 to 42.3 MPa. A 20% MRM addition effectively enhanced the mechanical properties of the mortar, while the mixture with 10% MRM and a 1:3 cement-to-sand ratio exhibited only 8.23% strength loss and 0.78% mass loss after 50 freeze–thaw cycles. MRM can improve the compactness of the cement matrix and thus optimize its freeze–thaw resistance, providing an eco-friendly technical solution for the engineering application of recycled mortar in cold regions.

## 1. Introduction

As the “adhesive” in construction, mortar serves to connect various building components, ensuring the stability and durability of the structure. It plays a crucial role in construction projects. With the advancement of construction technology and the growing emphasis on sustainable development principles, research aimed at improving mortar performance has become a central focus in the construction industry [1,2]. Recently, the goals of “Carbon Peaking and Carbon Neutrality” have generated significant interest in the resource utilization of construction solid waste within the industry [3,4].

In this context, promoting the green and low-carbon development of sustainable buildings by replacing natural sand with recycled fine aggregate (RFA) derived from construction waste, and using recycled micropowder (RM) as a substitute for traditional cement, is of great significance [5,6]. RM is a fine powder with a typical particle size of ≤0.075 mm, which is generated as a by-product during the crushing, screening, and mechanical processing of construction and demolition waste into recycled aggregates. It is primarily derived from waste concrete and clay bricks, and is classified into concrete-based recycled micropowder (CRM) and brick-based recycled micropowder (BRM) according to its raw material origin [7,8]. The physicochemical properties of RM vary significantly depending on the parent material, service age, exposure history, and carbonation degree of the source waste [9]. These variations directly influence pozzolanic activity, CaO content, particle morphology, and water demand, thereby affecting hydration behavior, pore refinement, and mechanical performance within cementitious systems [10]. For instance, CRM generally exhibits higher CaO content and greater mass loss during thermal treatment than BRM, making it more responsive to high-temperature calcination activation [11].

Incorporating mineral admixtures such as RM has been shown to improve mortar properties. These admixtures enhance flow, reactivity, and micro-aggregate filling effects, thereby optimizing mortar performance [12,13]. Although less reactive than fly ash in terms of pozzolanic activity, the high CaO content of RM effectively promotes hydration in the cement matrix [14]. Liu et al. [15] employed Mercury Intrusion Porosimetry (MIP) to quantify the impact of RM on pore structure, finding no significant increase in the number of harmful pores at a 25% substitution rate, but a notable rise when the content exceeded 50%. Therefore, an appropriate dosage of RM does not degrade mortar properties by increasing harmful pores. From a mechanical perspective, Su et al. [16] observed that cement mortar incorporating CRM exhibited a compressive strength approximately 36% higher than that of BRM, with performance comparable to fly ash. These findings underscore the significant potential of RM to enhance building material performance, demonstrating its feasibility and advantages as a viable alternative to traditional cement-based materials. However, RM inevitably contains organic impurities from mixed domestic waste and construction residues, which delays cement hydration, increases water demand and reduces matrix durability [17]. Effective removal of these impurities is crucial for improving the pozzolanic activity of RM.

Owing to the potential application of RM in cementitious materials, research has focused on activating its pozzolanic activity via chemical and physical activation methods [18]. The goal is to optimize its hydration behavior and microstructure within the cement matrix, thereby improving overall material performance. Chen et al. [19] demonstrated the advantages of synergistically combining chemical and thermal activation of RM. With a 30% RM admixture, the activity index for thermally activated RM was 11.6% higher than that with chemical activation alone. Zhang et al. [20] investigated the mechanical activation of RM, finding that 75 min of mechanical milling significantly enhanced its pozzolanic activity, contributing to improved compressive strength in the cement matrix. Activated RM clearly exhibits greater enhancement in cementitious materials compared to untreated RM. This comparison highlights the critical role of activation technology in enhancing material properties, establishing RM’s role as a high-performance concrete admixture.

Scholars from various regions [21,22] have investigated the use of crushed construction and demolition waste in the production of recycled mortar. However, the composition of construction and demolition waste exhibits notable regional heterogeneity due to differences in local building practices, economic development, and dominant construction materials [23]. Consequently, the quality of RFA and the resulting mortar can vary considerably across regions. In many cases, the particle size distribution of RFA is poor, and its surface is rich in old mortar with high porosity. When used as a substitute for natural sand, it adversely affects the workability, mechanical properties, and durability of the mortar [24,25]. Recycled mortar is widely used in road repair, agricultural engineering, and 3D printing component design. It improves construction quality and efficiency while promoting environmental sustainability and cleaner production [26,27]. Due to variations in the composition of construction waste across different regions, a detailed study was conducted on construction waste in a specific area of Qingdao. This waste was recycled and sieved into RM and RFA. The changes in functional groups and the thermal stability of the RM were analyzed in detail using thermogravimetric analysis (TGA), activation indicators, and Fourier transform infrared spectroscopy (FTIR). After high-temperature calcination of the CRM, recycled cement-based material (RCM) was prepared. The formation of hydration gels, pore structure evolution, and hydration kinetics in the cement matrix were thoroughly analyzed using X-ray diffraction (XRD) and Brunauer–Emmett–Teller (BET) methods. Finally, by varying the type and dosage of micropowder, as well as the cement-to-sand ratio (C/S), the water consumption, delamination degree, air content, compressive strength, and freeze–thaw resistance of recycled siliceous mortar (RSM) were tested and evaluated. The efficiency coefficient (K_λ_) was adopted to quantitatively evaluate the effects of FA, CRM, and modified recycled micropowder (MRM) on recycled mortar within this study. This approach quantitatively evaluates admixture effects in the studied system to support mix optimization.

## 2. Materials and Methods

### 2.1. Raw Materials

The experimental procedure utilized Ordinary Portland Cement (OPC), Grade P·O 42.5, with initial and final setting times of 204 and 334 min, respectively. The FA used was Grade II and sourced from a power plant in Qingdao. The RM was classified into two types: CRM and BRM. The chemical compositions and basic properties of the test materials are summarized in Table 1 and Table 2. Recycled fine aggregate (RFA) was classified as Type II fine aggregate and was produced by processing discarded concrete from a specific area in Qingdao. The basic physical properties of the RFA are shown in Table 3.

### 2.2. Preparation Method

#### 2.2.1. MRM Preparation Process

**Figure 1:**
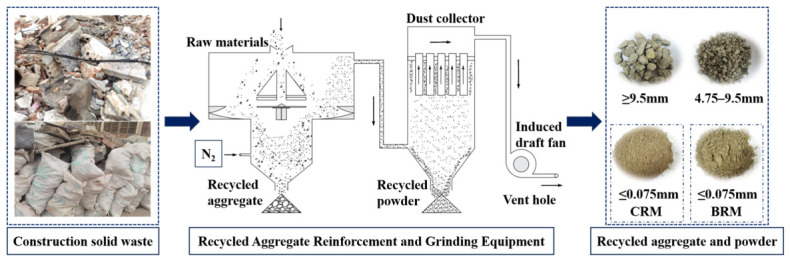
Preparation process of RM and RFA.

**Figure 2:**
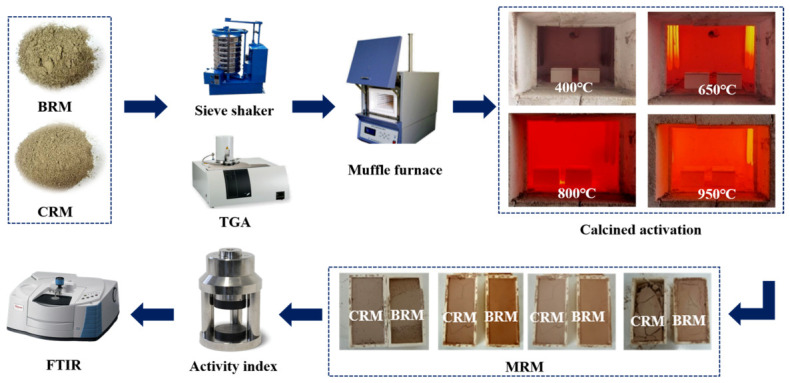
Activation and testing procedure of RM.

The construction solid waste was derived from discarded concrete and bricks. It was initially crushed to reduce its size, then processed in the “Recycled Aggregate Reinforcement and Grinding Equipment” for repeated shaping and strengthening of the recycled aggregates. The product was subsequently sieved. Through continuous refinement, the geometric characteristics of the RFA and RM were optimized. Finally, the RM was collected, sieved, and ball-milled at 300 r/min for 30 min with a ball-to-powder ratio of 5:1 to ensure compliance with the quality standards outlined in “Recycled Fine Aggregate for Concrete and Mortar” (GB/T 25176-2010) [28]. The preparation process for RM and RFA is illustrated in Figure 1. A portion of the RM with a particle size of ≤0.075 mm was first taken for thermogravimetric analysis (TGA). The TGA tests were performed using a Netzsch STA 449F3 thermogravimetric analyzer (Netzsch Instruments, Selb, Germany). Each sample was heated from room temperature to 800 °C at a constant heating rate of 10 °C/min. The mass loss and derivative thermogravimetric (DTG) curves were continuously recorded throughout the heating process. Subsequently, the remaining RM was placed in a muffle furnace for activation calcination. The samples were heated at a constant rate of 10 °C/min to the target temperatures of 400 °C, 650 °C, 800 °C, and 950 °C, respectively, and held at each target temperature for 2 h. All calcination processes were carried out in an air atmosphere. The samples were not quenched or forcibly cooled, but allowed to cool naturally to room temperature inside the closed muffle furnace. After cooling, the resulting MRM was ground and dried. Strength activity analysis was then performed on the MRM. Based on the comprehensive results of the TGA and strength activity analysis, Fourier transform infrared spectroscopy (FTIR) tests were conducted on CRM samples. The specific experimental procedure is shown in Figure 2.

#### 2.2.2. Performance Comparison Between CRM and BRM

**Figure 3:**
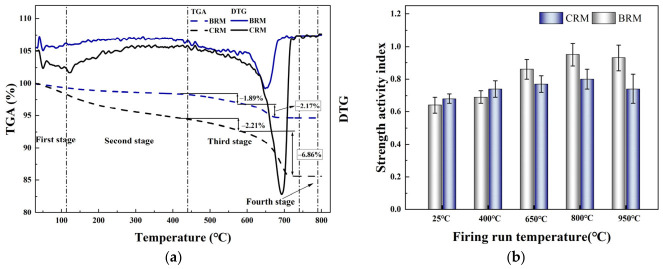
Comparison of CRM and BRM performance: (**a**) TGA of CRM and BRM; (**b**) strength activity index of CRM and BRM.

TGA was used to compare the performance changes in CRM and BRM after thermal activation. The thermal analysis data presented in Figure 3a show that both materials exhibited four distinct mass loss stages during calcination from 30 °C to 800 °C. The corresponding DTG curves further confirm that the third stage contributes the most to the total mass loss for both CRM and BRM, indicating that the primary thermal decomposition reactions occur within this temperature range. BRM showed moderate mass loss throughout the entire thermal process, with a cumulative loss of only 4.06% at 800 °C, indicating minimal structural changes upon heating. In contrast, CRM showed much more pronounced mass loss across all four stages, with a cumulative loss of 9.07% at 800 °C. This substantial difference in total mass loss confirmed that CRM underwent far more extensive thermal decomposition than BRM during calcination, which is mainly attributed to its higher content of calcium hydroxide, calcium silicate hydrate gel, and calcium carbonate [29,30]. As reported in previous thermal analysis studies of construction waste powders [31,32], this four-stage mass loss pattern is consistent with the thermal behavior of typical cement-based materials. The decomposition of these thermally unstable phases removes inert components and generates reactive species suitable for subsequent pozzolanic reactions [33,34].

The activity index presented in Figure 3b is the strength activity index determined in accordance with the method specified in JG/T 573-2020 “Recycled Fine Powder Used in Concrete and Mortar” [35]. Figure 3b presents the strength activity indices of CRM and BRM at various calcination temperatures. At 25 °C, both materials exhibited similar activity levels. As the calcination temperature increased to 400 °C, the activity of CRM rose to 0.75, slightly exceeding that of BRM, reflecting the initial activation effect of CRM through low-temperature calcination. When the temperature reached between 650 °C and 950 °C, BRM showed a higher peak activity, but its activity was more volatile. In contrast, CRM maintained a relatively stable activity level at high temperatures, indicating superior reaction stability under thermal stress. Notably, high-temperature treatment above 650 °C significantly enhanced the strength of CRM. Specifically, at 800 °C, the strength of CRM increased by 32.6%, and further increasing the temperature to 950 °C resulted in a 31.2% strength increase. Furthermore, the standard deviation at 950 °C exceeded 0.9, which was 41.6% higher than that at 800 °C. This may be due to the acceleration of the reaction rate at high temperatures, leading to an uneven distribution of active phases [36]. In contrast, CRM treated at 800 °C exhibited more favorable reaction kinetics.

**Figure 4:**
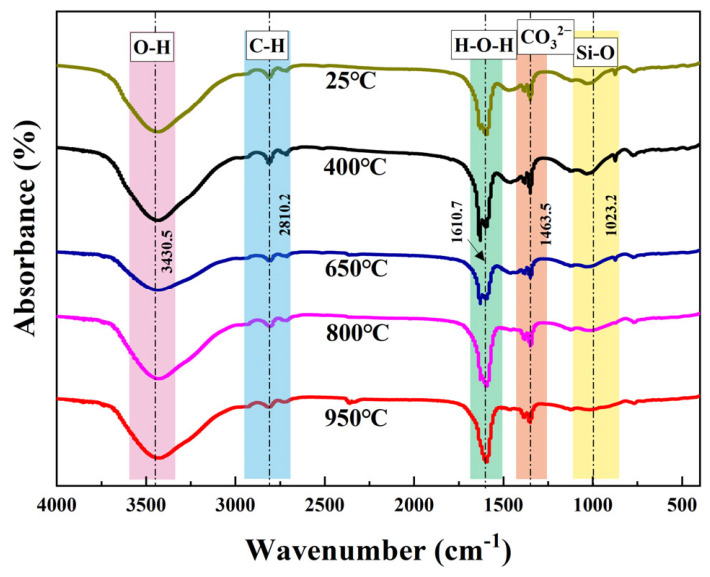
FTIR analysis of CRM at different calcination temperatures.

Figure 4 presents the FTIR analysis of CRM at different calcination activation temperatures. All key characteristic peaks and their corresponding chemical bonds have been annotated directly on the spectra. For clearer reference, Table 4 summarizes all main FTIR absorption bands and their assignments for CRM across different calcination temperatures. At 400 °C, the carbon-hydrogen peak associated with organic impurities weakened relative to the 25 °C sample but remained detectable. At 650 °C, most readily degradable organic components had decomposed, yet the carbonate peak at 1463 cm^−1^ observed in the uncalcined sample remained relatively prominent [37]. When the temperature reached 800 °C, the H-O-H bending vibration peak at 1610 cm^−1^ significantly weakened [38], indicating more thorough water removal. Concurrently, the intensity of the organic impurity peak near 2810 cm^−1^ decreased substantially, confirming the removal of most readily decomposable organic contaminants. Most importantly, the intensity and shape of the silicon–oxygen bond stretching vibration peak near 1022 cm^−1^ became more regular, which is consistent with the activation and reconstruction of the amorphous silicate–aluminate phase in CRM and correlates with the observed enhancement in pozzolanic activity [39,40]. Although the main decarbonation of CaCO_3_ typically occurs between 600 and 800 °C, residual carbonate bands persisted even at 950 °C. This is attributable to the heterogeneous nature of the recycled powder, in which some CaCO_3_ is encapsulated within particle cores or exists in more thermally stable forms, resulting in incomplete decomposition; minor re-carbonation may also occur during cooling in air [17]. Meanwhile, the partial crystallization of the active phase caused some deformation in the peak shape of the silicon–oxygen bonds, reducing their potential for activation [41]. The very weak residual peak near 2810 cm^−1^ at 950 °C is attributed to a small amount of refractory organic components, such as aromatic compounds, which cannot be completely decomposed below 950 °C [42].

Based on comprehensive analysis of the TGA, strength activity index, and FTIR, it can be concluded that calcination at 800 °C effectively removes most readily decomposable organic impurities, decomposes a large portion of carbonate phases, and optimally activates the amorphous silicate–aluminate phase in CRM. Therefore, this study selected the CRM sample activated at 800 °C for subsequent experiments.

### 2.3. Sample Preparation and Mix Proportion Design

#### 2.3.1. RCM and RSM Preparation Process

The preparation of RCM began with dry-mixing OPC and MRM for 90 s. A predetermined amount of water was then added and mixed. Once the RCM flow met the test requirements, as verified using a cement flow tester, it was poured into 40 mm × 40 mm × 40 mm molds and cured in water.

For RSM preparation, the dry raw materials including OPC, mineral admixture, and RFA were placed in a mixing pot and mixed for 180 s. Water was then added and mixed for an additional 60 s. After using the mortar consistency meter to confirm that the RSM met the test requirements, the cube mortar test blocks of 70.7 mm × 70.7 mm × 70.7 mm were cast. The test blocks were demolded after molding and standard-cured for 28 days under conditions of 20 ± 2 °C and a relative humidity of ≥95%.

#### 2.3.2. Mix Proportion Design of RCM and RSM

RCM was prepared by using RM as an admixture and replacing 20% of OPC by mass. The various properties of RCM were evaluated according to the “Fly Ash for Cement and Concrete” (GB/T 1596-2017) standard [43]. The specific mix ratio design is presented in Table 5.

**Figure 5:**
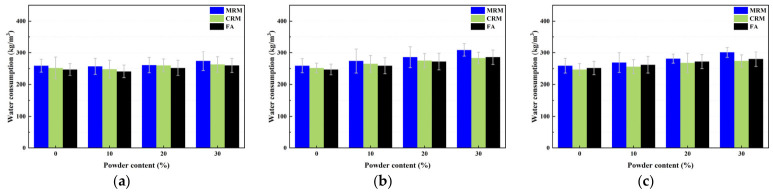
Water consumption of RSM: (**a**) C/S: 1/3, (**b**) C/S: 1/4, and (**c**) C/S: 1/5.

All mortar mix proportions in this study were strictly designed in accordance with the “Specification for Mix Proportion Design of Masonry Mortar” (JGJ/T 98-2010) [44]. All recycled fine aggregates were used in saturated surface-dry state as specified in the “Standard for Technical Requirements and Test Method of Sand and Crushed Stone (or Gravel) for Ordinary Concrete” (JGJ 52-2006) [45]. Three C/S ratios were selected: 1:3, 1:4, and 1:5. The mineral admixtures used included FA, CRM, and MRM (800 °C). Four proportions of mineral admixtures were incorporated: 0, 10%, 20%, and 30% of the total cementitious material mass. The water consumption for RSM was determined according to the “Standard for Test Method of Performance on Building Mortar” (JGJ/T 70-2009) [46]. The dosage of the naphthalene high-efficiency water reducer was set at 0.6% of the cementitious material mass. Mortar water retention was maintained at a minimum of 84% by adjusting the water consumption, and consistency was controlled between 70 and 90 mm. The specific mix ratios and water consumption are provided in Table 6 and Figure 5, respectively.

### 2.4. Test Methods

#### 2.4.1. XRD Test of RCM

After 28 days of standard water curing, RCM samples were gently crushed, immersed in acetone for 24 h to stop hydration, vacuum-dried at 40 °C for 48 h, and ground to pass a 75 μm sieve. XRD patterns were obtained using a Bruker D8 Advance diffractometer (Bruker AXS SE, Karlsruhe, Germany) with Cu Kα radiation, operated at 40 kV and 40 mA. Scans were performed over a 2θ range of 5–90° with a step size of 0.02° and a scan speed of 1°/min. Phase identification was performed using the ICDD PDF-4 database.

#### 2.4.2. Hydration Heat Test of RCM

In accordance with the mix proportions specified in Table 5, two paste mixtures incorporating CRM and MRM800, respectively, were prepared, and their hydration characteristics were characterized. The evolution of hydration heat was measured using a TAM Air 8-channel isothermal calorimeter (TA Instruments, New Castle, DE, USA) at a constant temperature of 20 ± 0.1 °C. Each batch of CRM or MRM800 was dry-mixed with OPC for 90 s, followed by the addition of water and further mixing for 60 s. Immediately after mixing, 5.00 ± 0.01 g of the test sample was loaded into each channel, with deionized water of equal mass used as the inert reference sample. Heat flow and cumulative heat release data were collected continuously for 72 h, with a data acquisition interval of 10 s.

#### 2.4.3. Macroscopic Performance Test of RSM

According to the industry standard “Standard for Test Method of Basic Properties of Construction Mortar” (JGJ/T 70-2009) [46], the water consumption, delamination degree, air content, mechanical properties, and freeze–thaw resistance of RSM were determined. All macroscopic performance tests were performed in triplicate, and the results were expressed as the mean value of three parallel specimens.

Delamination degree determination: The mortar was loaded into the delamination cylinder on the vibration table and vibrated for 20 s. Then, 100 mm of mortar was then removed from the bottom, mixed in an agitator for 2 min, and the consistency was measured again. The difference between the two measurements was recorded as the delamination degree (accurate to 1 mm).

Air content determination: The stirred mortar was loaded into the measuring pot in three layers, with the probe inserted into each layer several times. After cleaning the surface, the pot was sealed and pressurized using an air cylinder. Once the pointer stabilized, the reading was recorded as the air content. The air content of the mortar was determined as the average of two test results.

Mechanical property test: Cubic test blocks measuring 70.7 mm × 70.7 mm × 70.7 mm were fabricated for compressive strength testing. The loading rate was controlled between 0.25 and 1.5 kN/s.

Freeze–thaw resistance test: Specimens measuring 70.7 mm × 70.7 mm × 70.7 mm were fabricated, standard-cured for 28 days, and their initial conditions recorded. After saturation with water, the specimens were placed in a fully automatic freeze–thaw apparatus for testing. Following 50 freeze–thaw cycles (4 h of freezing and at least 4 h of thawing per cycle), the rates of strength loss and mass loss were calculated.

## 3. Results and Discussion

### 3.1. Performance Analysis of RCM

#### 3.1.1. Hydration Product Analysis of RCM

**Figure 6:**
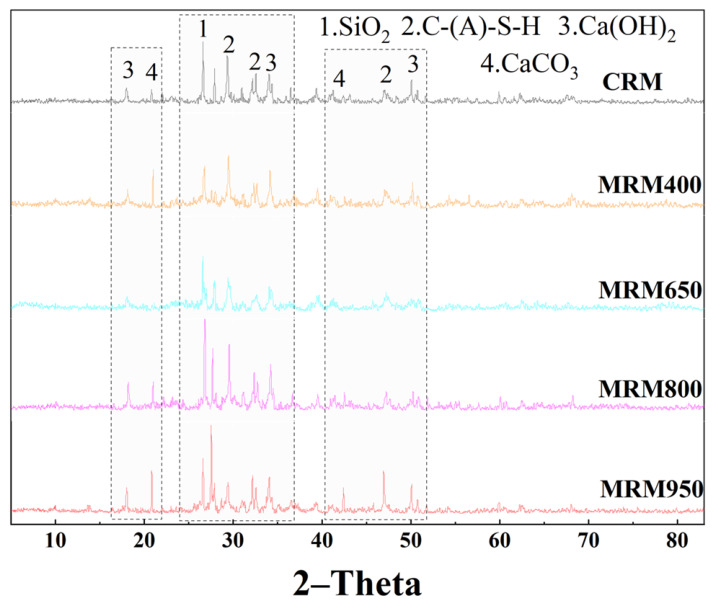
XRD patterns of 28-day hydration products of RCM with MRM calcined at different temperatures.

This study systematically investigated the 28-day hydration products of RCM prepared with MRM treated at different temperatures under standard curing conditions. Figure 6 shows the XRD patterns of hydration products of various RCM samples, with CRM-based RCM set as the reference group. At calcination temperatures of 400 °C and 650 °C, the broad diffraction features associated with poorly crystalline C-S-H in the corresponding RCM samples were relatively weak. In contrast, RCM incorporating MRM activated at 800 °C exhibited a more prominent broad diffraction feature corresponding to poorly crystalline C-S-H, as well as stronger characteristic peaks of Ca(OH)_2_. These observations are consistent with the thermal decomposition behavior of CRM phases at 800 °C, where C-S-H and clay minerals decompose to form amorphous reactive SiO_2_ and Al_2_O_3_, and CaCO_3_ and Ca(OH)_2_ decompose to form reactive CaO [47,48]. These active components subsequently participate in cement hydration and pozzolanic reactions, promoting the formation of additional C-S-H and C-A-S-H. Although MRM800 exhibits the highest pozzolanic activity, the higher Ca(OH)_2_ peak intensity is explained by the additional reactive CaO generated during calcination, which rapidly forms supplementary Ca(OH)_2_ upon hydration. At 28 days, the pozzolanic consumption of Ca(OH)_2_ had not yet fully offset this extra source, resulting in a net increase in Ca(OH)_2_ intensity while C-S-H formation was simultaneously enhanced [49]. Notably, when MRM was activated at 950 °C, the intensity of these hydrated product-related diffraction features in RCM decreased due to excessive calcination, which deteriorated the active phase of the micropowder.

#### 3.1.2. Hydration Heat Analysis of RCM

**Figure 7:**
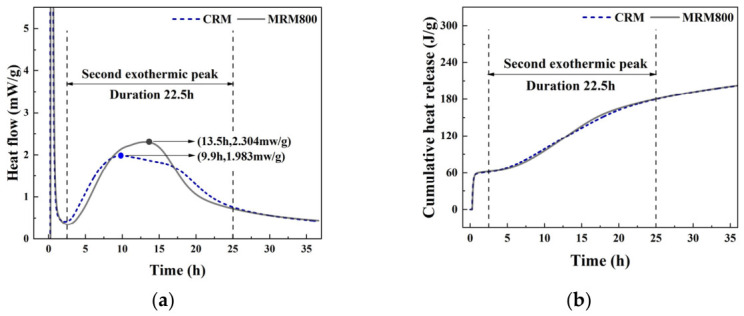
Hydration heat analysis of RCM. (**a**) Heat flow of CRM and MRM800; (**b**) Cumulative heat release for CRM and MRM800.

As shown in Figure 7a, the second exothermic peak of MRM800 reached its maximum at 13.5 h, with a heat flow intensity of 2.304 mW/g, which was higher than the peak intensity of CRM. This suggests that the hydration reaction of MRM800 is more intense at this stage. The reactive SiO_2_ and Al_2_O_3_ in MRM800 promoted the pozzolanic reaction, increasing heat release during the C_2_S hydration process, which in turn enhanced the formation of C-S-H and strengthened the second exothermic peak [50]. Figure 7b demonstrated that, during the test, the cumulative heat release of MRM800 was slightly higher than that of CRM, indicating a more complete hydration process for MRM800. This process decomposed some inert phases, generating more active cementitious components, such as the belite phase, which altered the mineral composition of MRM and thus enhanced the activity of the hydration reaction [51].

#### 3.1.3. Pore Structure Analysis of RCM

**Figure 8:**
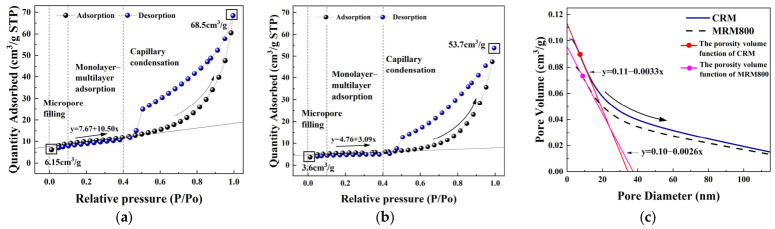
Pore structure analysis of RCM. (**a**) N_2_ adsorption/desorption isotherms of CRM, (**b**) N_2_ adsorption/desorption isotherms of MRM800, and (**c**) CRM and MRM on the change in RCM pore size.

Figure 8 illustrates changes in the pore structure of RCM. The N_2_ adsorption–desorption isotherms of RCM in Figure 8a,b conformed to IUPAC Type IV classification with H4-type hysteresis loops, which are typical characteristics of mesoporous cement-based materials. The CRM-based RCM had a wider adsorption platform in the micropore filling area, and the slope of the fitting equation was relatively large, indicating that the micropores on the CRM surface were dispersed and distributed [52,53]. These micropores are mainly caused by the presence of hydroxyl groups and residual adsorbed water in CRM. Its micropore adsorption capacity was 6.15 cm^3^/g, and the saturated adsorption volume was 68.5 cm^3^/g, both of which are higher than those of the MRM800-based RCM. In contrast, the micropore platform of the MRM800-based RCM is narrower because the adsorbed water was removed at 800 °C and the hydroxyl groups were transformed into active silicon/aluminum sites, which preferentially participated in secondary hydration rather than N_2_ adsorption; its hysteresis loop is more compact, indicating that the mesoporous network is more uniform. Complementing this, Figure 8c shows that MRM800-based RCM had a steeper pore volume decline in the 10–20 nm range, with a 42% lower volume here than in CRM-based RCM. This refinement stemmed from 800 °C calcination: it decomposed Ca(OH)_2_/CaCO_3_ into reactive CaO and elevated amorphous SiO_2_ content. These species reacted with Ca(OH)_2_ via pozzolanic reactions, generating additional C-S-H gel that filled micropores and partitioned large mesopores into small, discontinuous voids [54]. Comprehensive analysis primarily indicated that the active oxides in MRM affected the cement matrix’s microstructure, leading to internal pore densification and enhancing the overall material densification. It should be noted that BET/N_2_ adsorption only characterizes mesopores and cannot detect macropores critical to freeze–thaw durability, which is a limitation of this pore structure analysis.

#### 3.1.4. SEM of RCM

**Figure 9:**
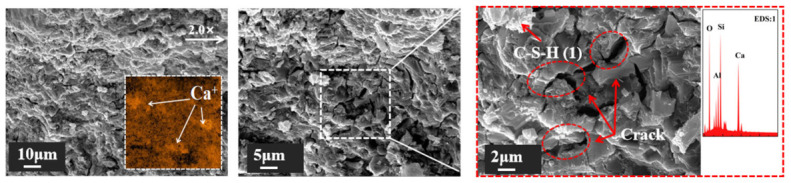
SEM of CRM-based RCM.

**Figure 10:**
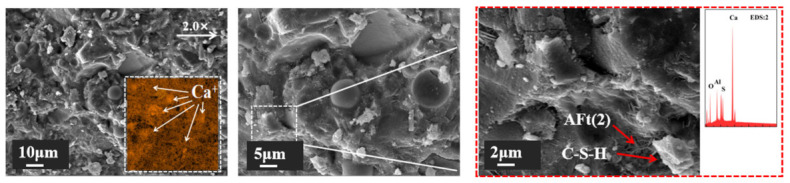
SEM of MRM800-based RCM.

The SEM images of RCM are shown in Figure 9 and Figure 10. As shown in Figure 9, the CRM-based sample exhibited a relatively porous microstructure with discrete microcracks and scattered C-S-H gel. EDS confirmed the presence of O, Si, Ca, and Al, and calcium ion mapping revealed a relatively dispersed distribution of Ca^2+^ [55]. In contrast, as shown in Figure 10, the MRM800-based sample showed a more concentrated Ca^2+^ distribution in its elemental mapping. Alongside C-S-H gel, needle-like AFt crystals were visible, and fewer microcracks were observed compared to the CRM-based reference. These morphological features were consistent with the increased formation of C-S-H and Ca(OH)_2_ observed in Figure 6, suggesting that MRM800 incorporation led to a more compact microstructure within the observed regions.

### 3.2. K_λ_ of RSM

Given the significant impact of mineral admixture type, admixture content, C/S ratio, and water consumption on the performance of RSM, this study introduced the efficiency coefficient K_λ_ to quantitatively evaluate the specific contributions of FA, CRM, and MRM to RSM performance. This section systematically clarifies the definition, physical meaning, theoretical basis, and evaluation criteria of the core parameters [43,56].

#### 3.2.1. Definition and Physical Meaning of Core Parameters

FA and CRM were used as control groups to more accurately reflect performance trends under multivariate influences. The core parameters for performance evaluation were defined as follows:

(1) β_f_: This parameter is the normalized performance index of RSM, calculated by the formula

(1) $$\beta_{f}=\frac{F_{c\lambda }}{F_{c0}}$$

where $F_{c\lambda }$ is the measured performance value of RSM with a mineral admixture substitution ratio of λ, λ is the mass ratio of the admixture to the total cementitious material, and $F_{c0}$ is the measured performance value of the blank RSM group without any mineral admixture. β_f_ quantifies the relative change in RSM performance after admixture addition, eliminating the influence of different C/S ratios on the absolute performance value.

(2) K_λ_: This core parameter quantifies the unit contribution efficiency of mineral admixtures, correcting the cement dilution effect caused by equal mass substitution. The calculation formula is:

(2) $$K_{\lambda }=\frac{\beta_{f}}{1-\lambda }.$$

This formula is based on the Inert Dilution Reference Hypothesis. When inert materials are replaced with equal amounts of cement under ideal linear conditions, the theoretical performance ratio of the system is 1 − λ [57,58]. This hypothesis is applicable in the 0–30% admixture substitution range of this study, verifying its rationality [59]. K_λ_ = 1 corresponds to the performance level of inert filler; K_λ_ > 1 indicates a positive contribution beyond inert dilution; K_λ_ < 1 indicates an adverse effect of the admixture [60,61].

#### 3.2.2. Evaluation Criteria and Uncertainty Analysis

The Aggregate Performance Critical Curve (APCC) was defined as the benchmark line of K_λ_ = 1, which was the critical threshold for distinguishing the positive and negative benefits of admixtures. It directly corresponds to the inert filler baseline, and can accurately judge whether the admixture meets the engineering application requirements of reducing cement consumption without performance degradation [15,62]. Considering the uncertainty of experimental tests, a systematic uncertainty analysis was carried out. The relative standard deviation (RSD) of all parallel test results was controlled within 8%, and the expanded uncertainty of K_λ_ was ±0.11. A conservative judgment criterion was adopted: K_λ_ > 1.11 indicated a significant positive benefit; K_λ_ < 0.89 indicated a significant negative effect; 0.89 ≤ K_λ_ ≤ 1.11 meant the performance was equivalent to inert filler. The corresponding formulas are summarized in Table 7.

### 3.3. Performance Evaluation of RSM

Masonry and plastering mortars are essential in construction, as they directly impact the quality, strength, service performance, and overall stability of brick masonry structures [63,64]. This chapter examines key performance indicators and engineering properties of RSM during both preparation and service, including water consumption, delamination degree, air content, mechanical properties, and freeze–thaw resistance.

#### 3.3.1. Water Consumption of RSM

**Figure 11:**
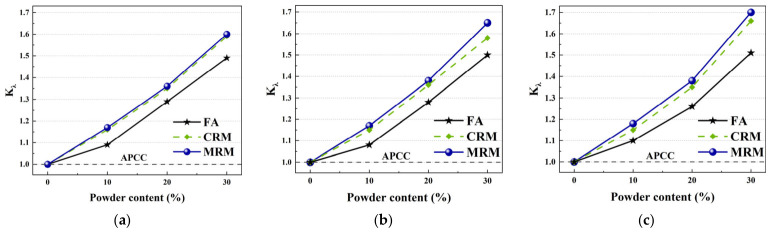
Evaluation of the water consumption of RSM using K_λ_: (**a**) C/S: 1/3, (**b**) C/S: 1/4, and (**c**) C/S: 1/5.

The water consumption of RSM at different C/S ratios was evaluated using the efficiency coefficient K_λ_, with the analysis presented in Figure 11. The results showed that water consumption increased with admixture dosage for all three materials. FA exhibited the most stable K_λ_ values across C/S ratios. All admixtures had K_λ_ > 1, reflecting a greater effect on water demand than inert fillers, mainly due to FA’s spherical, low-porosity particles [65]. CRM and MRM increased water demand due to higher specific surface area and powder characteristics. MRM showed a slightly higher K_λ_ than CRM, related to morphology changes after calcination. Consequently, although MRM increased water requirement more than an inert material, the K_λ_ framework confirmed that its net effect remained controllable within the 0–30% substitution range, providing a scientific basis for mix design adjustment.

#### 3.3.2. Delamination Degree of RSM

**Figure 12:**
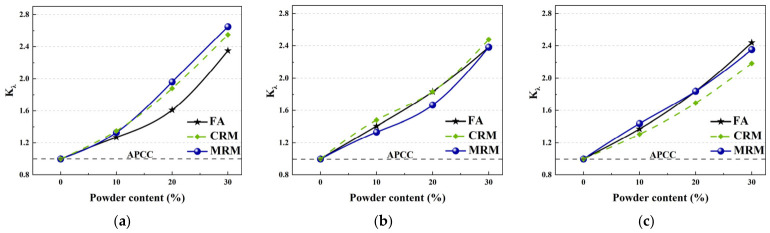
Evaluation of the delamination degree of RSM using K_λ_: (**a**) C/S: 1/3, (**b**) C/S: 1/4, and (**c**) C/S: 1/5.

The trends in the delamination degree of RSM at different C/S ratios were evaluated using the efficiency coefficient K_λ_, as shown in Figure 12. As the content of mineral admixtures increased, the K_λ_ for delamination degree exhibited an upward trend, meaning that the unit contribution of admixtures to increasing mortar delamination increased with dosage. Notably, MRM showed better overall performance than CRM in this regard: the K_λ_ for delamination degree of MRM-based RSM was 0.06–0.35 lower than that of CRM-based RSM at most mix ratios. Lower-density admixtures show higher buoyancy in water, causing surface enrichment. Weak inter-particle forces between filled admixtures and cement/aggregates also promote redistribution and higher delamination [66]. In contrast, the increased active CaO in MRM strengthened inter-particle interactions and the paste–aggregate interface, mitigating buoyancy-driven surface enrichment and reducing delamination tendency per unit mass more effectively than CRM. Consequently, MRM-based RSM exhibited improved homogeneity and construction adaptability, while reducing the risk of segregation during construction, thereby providing higher-quality materials for engineering projects.

#### 3.3.3. Air Content of RSM

**Figure 13:**
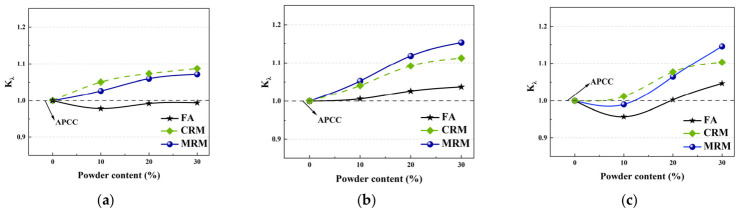
Evaluation of the air content of RSM using K_λ_: (**a**) C/S: 1/3, (**b**) C/S: 1/4, and (**c**) C/S: 1/5.

The air content of RSM at different C/S ratios was evaluated using the efficiency coefficient K_λ_, as shown in Figure 13. K_λ_ values generally increased with increasing mineral admixture dosage. Notably, several groups exhibited a K_λ_ < 1, demonstrating that these admixtures reduced air content per unit mass beyond the inert filler baseline. All three admixtures functioned primarily through physical filling, with their contribution efficiency showing a diminishing marginal effect as dosage increased. FA showed optimal filling from spherical vitreous particles. MRM’s efficiency was close to CRM’s and followed FA’s trend, refining pores at moderate dosages. Excessive MRM incorporation may disrupt normal cement hydration and lead to gas accumulation around particles. Therefore, K_λ_-guided dosage optimization based on the APCC line is critical for the precise control of air content and pore structure, which can further improve the workability, mechanical properties, and freeze–thaw resistance of recycled mortar.

#### 3.3.4. Mechanical Properties of RSM

**Figure 14:**
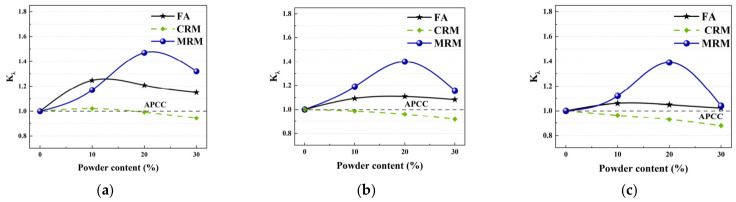
Evaluating the compressive strength of RSM using K_λ_: (**a**) C/S: 1/3, (**b**) C/S: 1/4, and (**c**) C/S: 1/5.

As shown in Figure 14, the mechanical properties of RSM at different cement-to-sand ratios were evaluated using the efficiency coefficient K_λ_ for compressive strength. The K_λ_ values for compressive strength showed an initial increase followed by a decrease with increasing FA and MRM dosage, indicating that their unit contribution to mechanical properties first increased and then decreased. RSM incorporating MRM exhibited improved performance, with compressive strength ranging from 24.3 to 42.3 MPa. This trend was consistent with the findings reported by Zhang et al. and Chen et al. [19,20]. MRM showed the highest unit efficiency, with a peak K_λ_ of 1.47 at 20% dosage. This value exceeded FA and the APCC baseline, indicating a notable positive contribution beyond inert dilution. This enhancement was attributed to the high content of reactive CaO and amorphous SiO_2_ in MRM, which promoted pozzolanic reactions and formed more C-S-H gel. In contrast, CRM showed a generally decreasing trend in compressive strength K_λ_ with increasing dosage. All CRM groups with a dosage of 10% and above had K_λ_ values less than 1, indicating that CRM could not offset the performance loss caused by cement dilution. Its contribution to mechanical properties was even lower than that of an inert filler, which was consistent with its low pozzolanic activity.

#### 3.3.5. Freeze–Thaw Resistance of RSM

**Figure 15:**
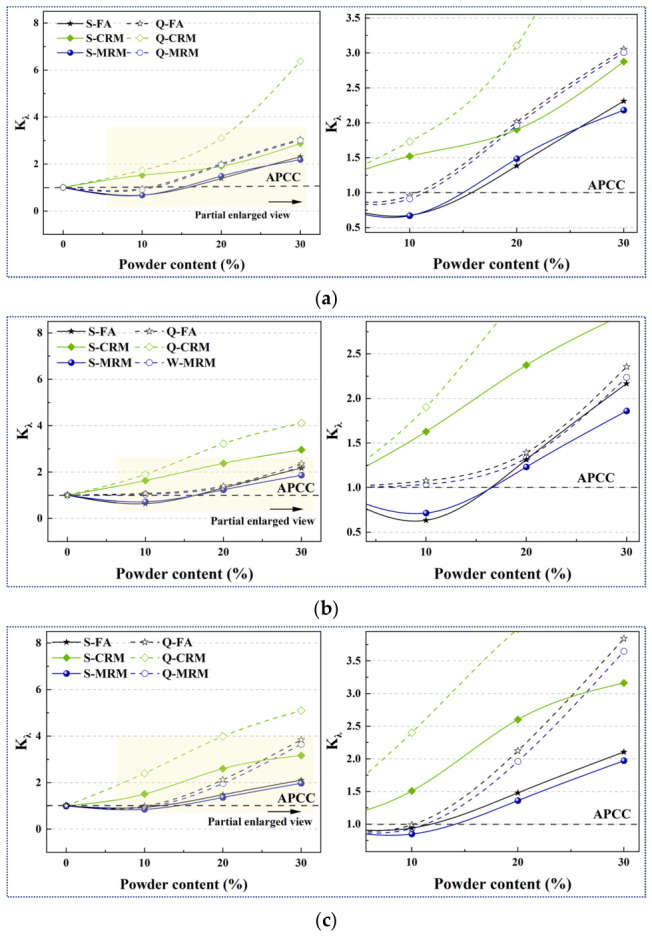
Evaluation of the freeze–thaw resistance of RSM using K_λ_: (**a**) C/S: 1/3, (**b**) C/S: 1/4, and (**c**) C/S: 1/5.

As shown in Figure 15 and Table 8, RSM samples standard-cured for 28 days were subjected to 50 freeze–thaw cycles to assess their strength loss rate and mass loss rate. The findings indicated that mortars containing FA or MRM initially exhibited a decreasing trend followed by an increase in strength loss rate, mass loss rate, and frost resistance K_λ_. Notably, RSM with 10% MRM and a 1:3 C/S ratio showed the lowest rates of strength loss and mass loss, corresponding to a frost resistance K_λ_ of 0.7, which was far below the APCC baseline. MRM per unit mass improved frost resistance beyond inert fillers, mainly by reducing large interconnected pores and limiting ice crystal expansion during freeze–thaw cycles. In contrast, all CRM groups with a dosage of 10% and above had frost resistance K_λ_ values greater than 1, indicating that CRM tended to deteriorate frost resistance per unit mass compared to an inert filler, which was consistent with its loose microstructure and low hydration activity [67]. In contrast to MRM, FA mainly improved the freeze–thaw resistance of mortar through pore structure refinement and water-binder ratio optimization created by its pozzolanic reactions and physical filling effect [68], while MRM achieved this improvement mainly through the chemical activation effect. High-temperature calcination enhanced its pozzolanic activity, promoted the generation of dense hydration products such as C-S-H gel, and thus optimized the pore structure of the matrix more directly. These results show that K_λ_ can quantitatively reflect freeze–thaw performance in this system.

## 4. Conclusions

This study systematically investigated the hydration product formation, pore structure evolution, and hydration kinetics of RM at different temperatures. FA, CRM, and MRM were used as mineral admixtures, and the efficiency coefficient K_λ_ was adopted to evaluate the workability, mechanical properties, and freeze–thaw resistance of RSM within the studied system. The main findings are as follows:

(1) TG and strength activity analyses of CRM and BRM show that CRM exhibited a mass loss of 9.07%, which was higher than BRM, and consistently greater strength activity at different temperatures. FTIR results further confirm that 800 °C is the optimal calcination temperature for CRM activation.
(2) XRD results indicate more pronounced characteristic signals of Ca(OH)_2_ and C-(A)-S-H in MRM800 compared with CRM. Hydration heat analysis reveals more intense secondary hydration and higher cumulative heat release for MRM800, indicating enhanced hydration activity. BET results show that MRM800 reduced nitrogen adsorption capacity by 21.6% compared with CRM, demonstrating a denser pore structure. These findings are consistent with SEM observations.
(3) K_λ_ evaluation of RSM workability shows that the water consumption K_λ_ increased with admixture dosage for all materials. At all C/S ratios, MRM increased the water consumption K_λ_ by 0.02–0.07 relative to CRM, but reduced the delamination K_λ_ by 0.06–0.35, indicating slightly higher water demand and improved homogeneity. A 10% MRM dosage reduced air content and refined pore structure.
(4) K_λ_ evaluation shows that 20% MRM effectively improved compressive strength at all C/S ratios, with K_λ_ exceeding the APCC baseline. After 50 freeze–thaw cycles, 10% MRM at a 1:3 C/S yielded optimal performance, while excessive MRM impaired freeze–thaw resistance.

Overall, recycled mortar containing 20% MRM exhibits compressive strength and freeze–thaw durability superior to conventional M15–M30 masonry mortar, fully meeting the requirements of mainstream engineering applications such as road bases, non-load-bearing masonry, and interior plastering.

## 5. Future Work

Future research will conduct long-term durability comparisons between fully recycled mortar and conventional natural sand mortar, and systematically elucidate the calcination activation mechanism of RM via quantitative XRD, portlandite consumption measurement, and amorphous phase quantification. Mercury intrusion porosimetry and X-ray computed tomography will be adopted to comprehensively characterize the full pore size distribution, while dynamic modulus evolution, crack observation and post-freeze–thaw microstructural analyses will be performed to explore the freeze–thaw damage mechanism and establish quantitative links between pore structure and durability.

## Figures and Tables

**Table 1:** Chemical composition of raw materials.

Category	CaO	SiO_2_	Al_2_O_3_	Fe_2_O_3_	MgO	Na_2_O	Others
OPC	64.75	20.42	6.36	5.42	2.07	0.79	0.19
FA	5.31	50.51	33.66	4.23	0.17	1.36	4.76
CRM	17.23	53.70	15.46	8.35	3.72	0.15	1.39
BRM	12.72	63.36	18.34	3.76	1.65	0.03	0.14

**Table 2:** Basic properties of raw materials.

Basic Properties	OPC	FA	CRM	BRM
Fineness (%)	4.3	20.6	22.2	21.8
Bulk density (kg/m^3^)	1398	1230	987	920

**Table 3:** Basic physical properties of the RFA.

Fineness Modulus	Apparent Density (kg/m^3^)	Bulk Density (kg/m^3^)	Porosity (%)	Water Absorption (%)	Fine Powder Content (%)	Soil Content (%)
2.8	2483	1342	41	7.6	0.7	0.75

**Table 4:** Main FTIR absorption bands and their assignments for CRM at different calcination temperatures.

Wavenumber (cm^−1^)	Chemical Bond/Functional Group
~3430.5	O–H stretching; portlandite and bound water
~2810.2	C–H stretching; organic impurities
~1610.7	H–O–H bending; physically bound water
~1463.5	CO_3_^2−^ asymmetric stretching; carbonate
~1023.2	Si–O stretching; silicate

**Table 5:** Mixing ratio design for RCM.

Group	Binding Material (kg/m^3^)	OPC (kg/m^3^)	Mineral Admixture	Admixture Dosage (kg/m^3^)	W/C (kg/m^3^)	Firing Run Temperature (°C)
CRM	400	320	CRM	80	0.31	–
MRM400	400	320	MRM	80	0.31	400
MRM650	400	320	MRM	80	0.31	650
MRM800	400	320	MRM	80	0.31	800
MRM950	400	320	MRM	80	0.31	950

Note: The RCM prepared from the CRM that had not undergone high-temperature calcination serves as the control group in the experiment. Among them, MRM400, MRM650, MRM800 and MRM950 correspond to MRM calcined at 400 °C, 650 °C, 800 °C, and 950 °C, respectively. W/C represents the water-to-cement ratio.

**Table 6:** Mixing ratio design for RSM.

Group	C/S	Binding Material (kg/m^3^)	OPC (kg/m^3^)	Mineral Admixture	Admixture Dosage (%)	RFA (kg/m^3^)
AF0	1:3	425	425.0	FA	0	1275
AF1	1:3	425	382.5	FA	10	1275
AF2	1:3	425	340.0	FA	20	1275
AF3	1:3	425	297.5	FA	30	1275
AR0	1:3	425	425.0	CRM	0	1275
AR1	1:3	425	382.5	CRM	10	1275
AR2	1:3	425	340.0	CRM	20	1275
AR3	1:3	425	297.5	CRM	30	1275
AM0	1:3	425	425.0	MRM	0	1275
AM1	1:3	425	382.5	MRM	10	1275
AM2	1:3	425	340.0	MRM	20	1275
AM3	1:3	425	297.5	MRM	30	1275
BF0	1:4	340	340.0	FA	0	1360
BF1	1:4	340	306.0	FA	10	1360
BF2	1:4	340	272.0	FA	20	1360
BF3	1:4	340	238.0	FA	30	1360
BR0	1:4	340	340.0	CRM	0	1360
BR1	1:4	340	306.0	CRM	10	1360
BR2	1:4	340	272.0	CRM	20	1360
BR3	1:4	340	238.0	CRM	30	1360
BM0	1:4	340	340.0	MRM	0	1360
BM1	1:4	340	306.0	MRM	10	1360
BM2	1:4	340	272.0	MRM	20	1360
BM3	1:4	340	238.0	MRM	30	1360
CF0	1:5	283	283.0	FA	0	1417
CF1	1:5	283	254.7	FA	10	1417
CF2	1:5	283	226.4	FA	20	1417
CF3	1:5	283	198.1	FA	30	1417
CR0	1:5	283	283.0	CRM	0	1417
CR1	1:5	283	254.7	CRM	10	1417
CR2	1:5	283	226.4	CRM	20	1417
CR3	1:5	283	198.1	CRM	30	1417
CM0	1:5	283	283.0	MRM	0	1417
CM1	1:5	283	254.7	MRM	10	1417
CM2	1:5	283	226.4	MRM	20	1417
CM3	1:5	283	198.1	MRM	30	1417

Note: A, B, and C respectively represent the C/S ratios of 1/3, 1/4, and 1/5. F represents FA. R represents CRM. M represents MRM. 0, 1, 2, and 3 respectively represent the content of mineral admixtures as 0, 10%, 20%, and 30%.

**Table 7:** Formulas for RSM.

	RSM Performance Ratio	Efficiency Coefficient	APCC
Formula	$\beta_{f}=\frac{F_{c\lambda }}{F_{c0}}$	$K_{\lambda }=\frac{\beta_{f}}{1-\lambda }$	y = 1

Note: β_f_ represents the measured performance ratio of RSM. F_cλ_ represents the performance measurement of RSM with admixtures. F_c0_ represents the measured performance of RSM without additives. K_λ_ is the efficiency coefficient, which depends on the additive content. λ represents the ratio of admixture mass to cementing material mass.

**Table 8:** Strength loss rate and mass loss rate of RSM.

Group	Strength Loss Rate (%)	Mass Loss Rate (%)	Group	Strength Loss Rate (%)	Mass Loss Rate (%)	Group	Strength Loss Rate (%)	Mass Loss Rate (%)
AF0	13.58	0.95	AR0	13.58	0.95	AM0	13.58	0.95
AF1	10.27	0.82	AR1	18.60	1.48	AM1	8.23	0.78
AF2	15.04	1.53	AR2	20.67	2.36	AM2	16.14	1.49
AF3	21.98	2.03	AR3	27.34	4.24	AM3	20.73	2.00
BF0	18.67	2.53	BR0	18.67	2.53	BM0	18.67	2.53
BF1	10.64	2.45	BR1	27.36	4.33	BM1	12.01	2.36
BF2	19.62	2.82	BR2	35.44	6.52	BM2	18.39	2.69
BF3	28.32	4.17	BR3	38.64	7.28	BM3	24.30	3.96
CF0	23.62	3.17	CR0	23.62	3.17	CM0	23.62	3.17
CF1	19.97	2.82	CR1	32.03	6.85	CM1	18.03	2.66
CF2	27.94	5.38	CR2	49.12	10.12	CM2	25.69	4.97
CF3	34.79	8.52	CR3	52.25	11.31	CM3	32.58	8.09

Note: A, B and C respectively represent the C/S ratios of 1/3, 1/4, and 1/5. F represents FA. R represents CRM. M represents MRM. 0, 1, 2, and 3 respectively represent the content of mineral admixtures as 0, 10%, 20%, and 30%.

## Data Availability

The original contributions presented in this study are included in the article. Further inquiries can be directed to the corresponding authors.

## Abbreviations

The following abbreviations are used in this manuscript:

AFt	Ettringite
APCC	Aggregate performance critical curve
BET	Brunauer–Emmett–Teller
BRM	Brick-based recycled micropowder
CRM	Concrete-based recycled micropowder
C/S	Cement-to-sand ratio
C-S-H	Calcium silicate hydrate
FA	Fly ash
FTIR	Fourier transform infrared spectroscopy
MIP	Mercury intrusion porosimetry
MRM	Modified recycled micropowder
OPC	Ordinary Portland cement
RCM	Recycled cement-based material
RFA	Recycled fine aggregate
RM	Recycled micropowder
RSM	Recycled silica-based mortar
TGA	Thermogravimetric analysis
XRD	X-ray diffraction
W/C	Water-to-cement ratio
Δfm	Strength loss of RSM
Δmm	Mass loss of RSM
β_f_	RSM performance ratio
F_cλ_	The performance value of RSM with a mineral admixture substitution ratio of λ
F_c0_	The performance value of the blank RSM group without any mineral admixture
